# The interaction of adverse childhood experiences, sex, and transgender identity as risk factors for depression: disparities in transgender adults

**DOI:** 10.3389/fgwh.2024.1306065

**Published:** 2024-12-24

**Authors:** Siva Balakrishnan, Wei Yang, Ann M. Weber

**Affiliations:** School of Public Health, University of Nevada, Reno, NV, United States

**Keywords:** gender, sex, adverse childhood experiences, depression, interaction, moderation, transgender, intersectionality

## Abstract

**Introduction:**

The intersectionality of sexism, transphobia, and adverse childhood experiences (ACE) on the mental health of transgender adults is poorly understood. We assessed whether the known association between ACE and depression was modified (or differed) by adult transgender identity and by assigned sex at birth, which we used as a proxy for adults' biological (e.g., hormonal changes) and social (e.g., sexism) experiences in childhood.

**Methods:**

Data from a representative sample of 519 transgender and 127,214 cisgender US adults was retrieved from the 2019 and 2020 Behavioral Risk Factor Surveillance System surveys. The sample was stratified by assigned sex at birth. Adjusted, sample-weighted Poisson regressions and relative excess risk due to interaction (RERI) were used to assess whether transgender identity modified the association between ACE and depression within strata of assigned sex at birth.

**Results:**

We found 42.4% of transgender compared to 24.9% of cisgender adults experienced 3+ ACE. The association between ACE and depression was stronger for transgender compared to cisgender adults, regardless of assigned sex at birth. However, transgender adults assigned female at birth with 1–2 ACE had a combined risk of depression that was higher than would be expected from the sum of the two risks alone (RERI test of interaction for transgender with 1–2 ACE vs. cisgender with zero ACE: 1.91 [95% confidence interval 0.47–3.36]; *p* = 0.009). The tests of interaction were not statistically significant (*p* > 0.05) for those assigned male at birth.

**Discussion:**

Transgender adults may benefit more from depression interventions informed by and addressing childhood trauma than their cisgender counterparts, particularly for transgender adults who were assigned female at birth. Our findings suggest a need for greater and improved data collection of gender, experiences associated with assigned sex at birth, and ACE as these relate to the transgender community. This will allow for a better understanding of the intersecting influences of sexism, transphobia, and ACE on adult depression and for identifying particularly vulnerable sub-populations in need of support.

## Introduction

Transgender individuals, or individuals whose gender identity does not align with their birth sex, are historically underrepresented in health research despite experiencing numerous challenges to their physical and mental health (1). Transgender adults have a higher prevalence of depression, anxiety disorder, post-traumatic stress disorder (PTSD), suicidal ideation, and substance abuse disorder than cisgender adults, or individuals whose gender identity aligns with their birth sex (2). These associations can be attributed to minority stress (i.e., stress experienced by individuals in stigmatized minority communities) and transphobia. For example, transgender children experience more social stigma, childhood abuse, and polyvictimization than their cisgender peers, all of which are associated with adulthood depression and PTSD (3). There is a need for further research to understand the extent of their health disparities.

Adverse childhood experiences (ACE) are associated with poor health outcomes in adulthood, including depression (4). ACE include household dysfunctions, such as divorce, familial mental illness, and household substance abuse, and abuses perpetrated against the child, such as sexual, physical, and verbal abuse. The higher the number of ACE an individual has experienced, the higher their risk of poor physical and mental health in adulthood (4).

Transgender adults have a higher prevalence of emotional neglect, emotional abuse, and having at least one ACE compared to cisgender adults (5, 6). As many as 92% of adult transgender men reported having experienced at least one ACE compared to 57.8% of cisgender adults (7, 8). This problem is likely to worsen as Generation Z (i.e., people born between 1997 and 2012) are more likely to identify as lesbian, gay, bisexual, transgender, queer, or questioning (LGBTQ+) and are at higher odds of experiencing 4+ ACE compared to older generations (9). While the literature on the effect of ACE on mental health in transgender populations is limited, evidence indicates that transgender men with four or more ACE have increased odds of suicide, depression, PTSD, and intimate partner violence (8). Furthermore, transgender children are at higher risk for abuse, putting them at risk for worse mental health as adults compared to their cisgender peers (5).

The effect of ACE on depression differs by sex assigned at birth, with females more negatively impacted by ACE than males (10). Similarly, gender is associated with ACE; feminine-identifying adults experience more childhood sexual abuse than masculine-identifying adults (11). Even within the transgender population, there are gender differences: for example, transgender women experience more social stigma than transgender men (12). Furthermore, biological sex is strongly associated with depression; females have a higher prevalence of biomarkers, such as genes and protein expression, associated with depression than males (13). However, conflation between sex and gender is common in public health research, so it is difficult to disentangle associations between ACE, gender, and sex (14, 15).

To our knowledge, no prior literature exists on whether the association between ACE and depression differs by transgender identity accounting for assigned sex. Prior studies on ACE in the transgender population had small samples that were not representative of the USA and did not examine differences in this association (i.e., test for effect modification) by gender. A large, representative study found stronger associations between ACE and depression among transgender compared to cisgender participants (16). However, the study did not account for possible interaction with assigned sex at birth when examining associations with ACE. Including assigned sex at birth is important because both biological and social components of assigned sex play a role in childhood experiences. For example, assigned sex influences the gender presentation of children and the behaviors of their parents and peers. Individuals assigned female at birth (AFAB) are more likely to experience sexism than individuals assigned male at birth (AMAB). Transgender men, despite identifying as men, can experience sexism during their childhood, which is associated with poor health outcomes (17). When children, including transgender children, go through puberty, they are influenced by hormones associated with their assigned sex at birth, unless they are prescribed puberty blockers. Puberty has been shown to more negatively affect mental health in children assigned female compared to children assigned male at birth (18). Sexism, transphobia, and ACE may interact to produce a greater impact on the mental health of transgender children compared to the effect of any one of these factors alone. Current public health literature is limited in the discussion of intersectionality (19). Efforts to address the mental health of transgender adults would benefit from an understanding of the intersectionality of sexism, transphobia, and ACE.

We aimed to identify whether the association between ACE and depression was modified by transgender identity in a representative sample of US adults. Given the strong effect of biological sex on the incidence of depression and to account for childhood experiences of sexism, we stratified the sample by assigned sex at birth, our proxy for those biological and social influences. We hypothesized that the association between ACE and depression would be greater for those who identified as transgender compared to cisgender and that this effect modification by transgender identity would hold regardless of adults’ experience of their assigned sex at birth.

## Methods

### Sample population

**Figure 1:**
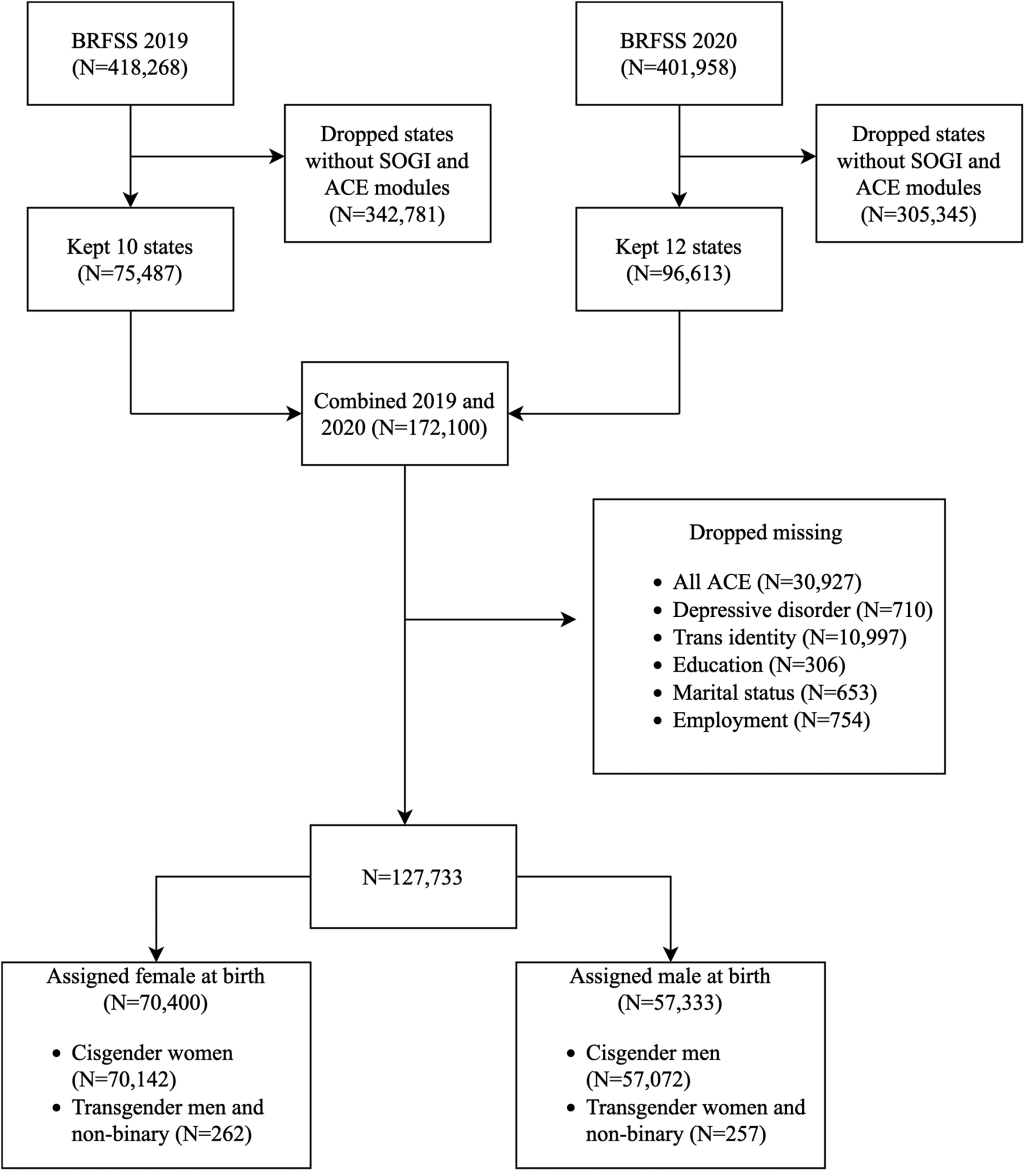
Flowchart showing the retrieval, cleaning, and merging of Behavior Risk Factor Surveillance System 2019 and 2020 datasets. Transgender identity and adverse childhood experiences information were retrieved from the sexual orientation and gender identity and adverse childhood experiences modules, respectively. ^a^BRFSS, Behavioral Risk Factor Surveillance System; ACE, adverse childhood experiences; SOGI, Sexual Orientation and Gender Identity.

We used nationally representative data collected by the 2019 and 2020 Behavioral Risk Factor Surveillance System (BRFSS) (20, 21). The BRFSS is an annual telephone survey of non-institutionalized US adults, monitoring chronic health conditions and risk behaviors. Surveyors record participants’ verbal consent after informing them of potential risks and conflicts of interest. The core survey is conducted in 50 states, the District of Columbia, and US territories, along with optional modules selected by each state. ACE and transgender questions were in optional modules, such that our final sample contained data from 16 states (Figure 1) (22, 23). As we used deidentified data, our study was exempt from institutional review.

### Measures

#### Exposure

The ACE module was developed for the CDC-Kaiser ACE study and has shown high test–retest reliability (24). It has 11 questions on household dysfunctions and abuses (Supplementary Table S1). In 2019, the ACE module was used in 17 states, and in 2020, it was used in 21 states and the District of Columbia.

The participants with “unsure,” “refused,” or missing responses to all ACE questions were removed (*n* = 30,927). Responses to the ACE questions were dichotomized (1 = present, 0 = not present, missing, unsure, or refused) (Supplementary Table S1). Sexual abuse was coded as present if the respondent answered “once” or more to any of the three sexual abuse questions. The participants were grouped by cumulative ACE scores (0, 1–2, and 3 or more ACE) to create the main exposure. These categories are frequently used in ACE research, originating from the CDC-Kaiser ACE study (24).

#### Outcome

The outcome was self-reported prior diagnosis of depression. Respondents were asked if they were ever told they had a depressive disorder, which included depression, major depression, minor depression, or dysthymia. This question was asked in all 50 states, the District of Columbia, and US territories. Responses were dichotomized as yes/no.

#### Transgender identity and sex at birth

The Sexual Orientation and Gender Identity (SOGI) module asked respondents about their gender identity. The participants could report being cisgender, transgender (which included transmen, transwomen, and non-binary adults), or unsure. In 2019 and 2020, the SOGI module was used in 31 and 33 states, respectively. We used a binary indicator (1 = transgender, 0 = cisgender) for transgender identity.

Data were stratified by assigned sex at birth. The core module asked the participants whether they were female or male, with no option for intersex. Because the sex question did not specify “at birth,” some transgender participants reported their gender identity instead of their sex. Therefore, assigned sex at birth for transgender men and women was manually entered as female and male, respectively. The assigned sex at birth of cisgender men, cisgender women, and transgender non-binary participants was kept as reported. For our analyses, we compared transgender adults assigned female at birth (AFAB) to cisgender AFAB adults and transgender adults assigned male at birth (AMAB) to cisgender AMAB adults. We use the AFAB and AMAB terminology instead of transgender/cisgender men and transgender/cisgender women because we included non-binary adults in our analyses. For example, the transgender AFAB adult group includes both transgender men and transgender non-binary AFAB adults.

#### Covariates

The covariates were age, race, and measures of socioeconomic position (SEP), which included marital status, employment, education, and income. We used BRFSS categories (25, 26) for age (18–24, 25–34, 35–44, 45–54, 55–64, >65 years), race/ethnicity (Hispanic and non-Hispanic White, Black, Asian, American Indian/Alaskan Native, and other), education (less than high school graduate, high school graduate, some college, and college graduate), and income (increments of $15,000 up to >$50,000). Marital status was reported as married, divorced, widowed, separated, never married, or member of an unmarried couple. For simplicity, we created a binary indicator for marital status (1 = married, 0 = not). Employment was grouped as employed, unemployed, and other, which included students and retirees.

### Missingness and imputation

States and territories that did not use the SOGI or ACE modules for 2019 or 2020 were excluded, leaving 172,100 observations from 16 states. Respondents missing depression (*n* = 710), transgender identity (*n* = 10,997), marital status (*n* = 653), employment (*n* = 754), or education (*n* = 306) were excluded. Many participants had missing annual household income information (*n* = 19,352) but were not excluded. Instead, age, race, education, marital status, employment, state, urban/rural status, and interview date were used to impute income groups using multinomial logistic regression. To account for the strong effect of sex on the incidence of depression, the dataset was stratified by assigned sex at birth. Among those assigned female at birth, there were 70,142 cisgender and 262 transgender adults. Among those assigned male at birth, there were 57,072 cisgender and 257 transgender adults.

### Statistical analysis

Data were analyzed using STATA 18 software (27). Sampling weights were assigned in accordance with standard BRFSS procedure (20, 21), reducing nonresponse bias and achieving a more representative sample. To test whether reported depression was associated with the COVID-19 pandemic, a logistic regression was run on depression adjusted for sex, age groups, and date of interview. A plot of the residuals of this regression by date of interview was created. The plot did not indicate a trend in diagnoses over time (plot not shown). Descriptive statistics of ACE, depression, transgender identity, and covariates were calculated for the full analytic sample and by strata. We used weighted Poisson regression to estimate the prevalence rate ratios (PRRs) of the effect of ACE on depression for each stratum, adjusting for age, race, and SEP variables. We estimated PRRs instead of odds ratios because odds ratios overestimate relative risk for common outcomes such as depression. Although ACE have been associated with adult SEP, parent SEP is also strongly associated with both adult SEP and ACE, confounding the relationship between ACE and depression (Supplementary Figure S1). Since we could not adjust for parent SEP, we adjusted for adult SEP and presented our results as the lower bound of the effect of ACE on depression. As a sensitivity check, we ran models adjusting only for age and race and found no significant differences in the interpretation of results (Supplementary Tables S2, S3).

The ratio of PRRs and relative excess risk due to interaction (RERI) methods were used to calculate multiplicative and additive effect measure modification (EMM), respectively. The RERI method calculates EMM on the additive scale using relative measures (28). Effect modification on the additive scale is particularly important because it can identify vulnerable subgroups that would benefit the most from health intervention, as well as identify pathways for new treatments to target. *P*-values less than 0.05 were deemed significant.

Additionally, we plotted predicted probabilities of depression for each stratum of ACE by gender identity to provide a visual representation of EMM (Supplementary Figure S2). Visual differences in the slopes of the trends for transgender and cisgender groups indicate significant EMM.

## Results

### Demographic, socioeconomic, ACE, and depression characteristics

Table 1 shows the demographic, socioeconomic, ACE, and depression characteristics of cisgender and transgender participants. Overall, 127,733 participants of the BRFSS 2019–2020 were included in the analysis, consisting of 519 transgender and 127,214 cisgender adults. Transgender participants had a higher prevalence of every individual ACE, three or more ACE, and depression compared to cisgender participants.

**Table 1:** Prevalence of demographic, socioeconomic, adverse childhood experiences, and depression characteristics of transgender and cisgender participants of the Behavioral Risk Factor Surveillance System 2019 and 2020 surveys.

	*n* (%)		
	Cisgender (*n* = 127,214)	Transgender (*n* = 519)	Total (*n* = 127,733)
Assigned sex			
Assigned female at birth	70,142 (55.1%)	262 (50.5%)	70,404 (55.1%)
Assigned male at birth	57,072 (44.9%)	257 (49.5%)	57,329 (44.9%)
Age group			
18–24	7,712 (6.1%)	96 (18.5%)	7,808 (6.1%)
25–34	12,682 (10.0%)	100 (19.3%)	12,782 (10.0%)
35–44	15,653 (12.3%)	71 (13.7%)	15,724 (12.3%)
45–54	18,759 (14.7%)	62 (11.9%)	18,821 (14.7%)
55–64	25,364 (19.9%)	64 (12.3%)	25,428 (19.9%)
65+	47,044 (37.0%)	126 (24.3%)	47,170 (36.9%)
Race			
Non-Hispanic White	97,751 (76.8%)	338 (65.1%)	98,089 (76.8%)
Non-Hispanic Black	11,102 (8.7%)	43 (8.3%)	11,145 (8.7%)
Non-Hispanic Asian	3,346 (2.6%)	19 (3.7%)	3,365 (2.6%)
Non-Hispanic American Indian/Alaskan Native	1,280 (1.0%)	11 (2.1%)	1,291 (1.0%)
Hispanic	8,843 (7.0%)	70 (13.5%)	8,913 (7.0%)
Non-Hispanic other	4,892 (3.8%)	38 (7.3%)	4,930 (3.9%)
Education			
Less than high school graduate	9,114 (7.2%)	72 (13.9%)	9,186 (7.2%)
High school graduate	34,742 (27.3%)	182 (35.1%)	34,924 (27.3%)
Some college	36,846 (29.0%)	128 (24.7%)	36,974 (28.9%)
College graduate	46,512 (36.6%)	137 (26.4%)	46,649 (36.5%)
Married			
No	60,366 (47.5%)	356 (68.6%)	60,722 (47.5%)
Yes	66,848 (52.5%)	163 (31.4%)	67,011 (52.5%)
Income group			
<$15,000	9,230 (7.3%)	64 (12.3%)	9,294 (7.3%)
$15,000–$24,999	16,680 (13.1%)	119 (22.9%)	16,799 (13.2%)
$25,000–$34,999	11,021 (8.7%)	62 (11.9%)	11,083 (8.7%)
$35,000–$49,999	15,192 (11.9%)	50 (9.6%)	15,242 (11.9%)
>$50,000	55,830 (43.9%)	133 (25.6%)	55,963 (43.8%)
Missing	19,261 (15.1%)	91 (17.5%)	19,352 (15.2%)
Employment			
Employed	62,707 (49.3%)	244 (47.0%)	62,951 (49.3%)
Unemployed	5,875 (4.6%)	51 (9.8%)	5,926 (4.6%)
Other	58,632 (46.1%)	224 (43.2%)	58,856 (46.1%)
Individual ACE			
Mental illness in household	20,904 (16.4%)	169 (32.6%)	21,073 (16.5%)
Alcoholic in household	28,516 (22.4%)	146 (28.1%)	28,662 (22.4%)
Substance abuser in household	12,019 (9.4%)	85 (16.4%)	12,104 (9.5%)
Parental divorce or separation	31,389 (24.7%)	167 (32.2%)	31,556 (24.7%)
Family member incarcerated	8,848 (7.0%)	77 (14.8%)	8,925 (7.0%)
Domestic violence in household	19,589 (15.4%)	116 (22.4%)	19,705 (15.4%)
Physical abuse	29,088 (22.9%)	172 (33.1%)	29,260 (22.9%)
Verbal abuse	40,542 (31.9%)	245 (47.2%)	40,787 (31.9%)
Sexual abuse	16,258 (12.8%)	104 (20.0%)	16,362 (12.8%)
ACE group			
0	48,989 (38.5%)	145 (27.9%)	49,134 (38.5%)
1–2	46,531 (36.6%)	154 (29.7%)	46,685 (36.5%)
3+	31,694 (24.9%)	220 (42.4%)	31,914 (25.0%)
Depression			
No	102,258 (80.4%)	298 (57.4%)	102,556 (80.3%)
Yes	24,956 (19.6%)	221 (42.6%)	25,177 (19.7%)

ACE, adverse childhood experiences.

Table 2 shows the demographic, socioeconomic, ACE, and depression characteristics of participants stratified by assigned sex at birth and transgender identity. Transgender AMAB participants had the highest prevalence of three or more ACE at 44%. Sexual abuse was most prevalent in transgender AFAB participants (22.9%), followed by cisgender AFAB adults (17.4%). Cisgender AFAB participants had a higher prevalence of depression compared to cisgender AMAB participants (24.2% vs. 14.0%). A similar, but smaller, difference in prevalence of depression was observed in transgender AFAB vs. transgender AMAB participants (45.0% vs. 40.1%).

**Table 2:** Prevalence of demographic, socioeconomic, adverse childhood experiences, and depression characteristics stratified by assigned sex at birth and transgender identity of participants of the Behavioral Risk Factor Surveillance System 2019 and 2020 surveys.

	*n* (%)			
	Assigned female at birth		Assigned male at birth	
	Cisgender (*n* = 70,142)	Transgender (*n* = 262)	Cisgender (*n* = 57,072)	Transgender (*n* = 257)
Age group				
18–24	3,515 (5.0%)	53 (20.2%)	4,197 (7.4%)	43 (16.7%)
25–34	6,494 (9.3%)	56 (21.4%)	6,188 (10.8%)	44 (17.1%)
35–44	8,441 (12.0%)	33 (12.6%)	7,212 (12.6%)	38 (14.8%)
45–54	10,131 (14.4%)	34 (13.0%)	8,628 (15.1%)	28 (10.9%)
55–64	13,947 (19.9%)	24 (9.2%)	11,417 (20.0%)	40 (15.6%)
65+	27,614 (39.4%)	62 (23.7%)	19,430 (34.0%)	64 (24.9%)
Race				
Non-Hispanic White	53,334 (76.0%)	165 (63.0%)	44,417 (77.8%)	173 (67.3%)
Non-Hispanic Black	6,940 (9.9%)	21 (8.0%)	4,162 (7.3%)	22 (8.6%)
Non-Hispanic Asian	1,763 (2.5%)	9 (3.4%)	1,583 (2.8%)	10 (3.9%)
Non-Hispanic American Indian/Alaskan Native	699 (1.0%)	7 (2.7%)	581 (1.0%)	4 (1.6%)
Hispanic	4,838 (6.9%)	38 (14.5%)	4,005 (7.0%)	32 (12.5%)
Non-Hispanic other	2,568 (3.7%)	22 (8.4%)	2,324 (4.1%)	16 (6.2%)
Education				
Less than high school graduate	4,893 (7.0%)	36 (13.7%)	4,221 (7.4%)	36 (14.0%)
High school graduate	18,624 (26.6%)	83 (31.7%)	16,118 (28.2%)	99 (38.5%)
Some college	21,227 (30.3%)	61 (23.3%)	15,619 (27.4%)	67 (26.1%)
College graduate	25,398 (36.2%)	82 (31.3%)	21,114 (37.0%)	55 (21.4%)
Married				
No	35,712 (50.9%)	177 (67.6%)	24,654 (43.2%)	179 (69.6%)
Yes	34,430 (49.1%)	85 (32.4%)	32,418 (56.8%)	78 (30.4%)
Income group				
<$15,000	5,854 (8.3%)	26 (9.9%)	3,376 (5.9%)	38 (14.8%)
$15,000–$24,999	10,245 (14.6%)	54 (20.6%)	6,435 (11.3%)	65 (25.3%)
$25,000–$34,999	6,409 (9.1%)	37 (14.1%)	4,612 (8.1%)	25 (9.7%)
$35,000–$49,999	8,255 (11.8%)	23 (8.8%)	6,937 (12.2%)	27 (10.5%)
>$50,000	27,212 (38.8%)	77 (29.4%)	28,618 (50.1%)	56 (21.8%)
Missing	12,167 (17.3%)	45 (17.2%)	7,094 (12.4%)	46 (17.9%)
Employment				
Employed	30,385 (43.3%)	119 (45.4%)	32,322 (56.6%)	125 (48.6%)
Unemployed	3,190 (4.5%)	29 (11.1%)	2,685 (4.7%)	22 (8.6%)
Other	36,567 (52.1%)	114 (43.5%)	22,065 (38.7%)	110 (42.8%)
Individual ACE				
Mental illness in household	13,242 (18.9%)	90 (34.4%)	7,662 (13.4%)	79 (30.7%)
Alcoholic in household	16,976 (24.2%)	72 (27.5%)	11,540 (20.2%)	74 (28.8%)
Substance abuser in household	6,610 (9.4%)	45 (17.2%)	5,409 (9.5%)	40 (15.6%)
Parental divorce or separation	17,384 (24.8%)	83 (31.7%)	14,005 (24.5%)	84 (32.7%)
Family member incarcerated	4,676 (6.7%)	34 (13.0%)	4,172 (7.3%)	43 (16.7%)
Domestic violence in household	11,453 (16.3%)	57 (21.8%)	8,136 (14.3%)	59 (23.0%)
Physical abuse	15,435 (22.0%)	78 (29.8%)	13,653 (23.9%)	94 (36.6%)
Verbal abuse	22,302 (31.8%)	120 (45.8%)	18,240 (32.0%)	125 (48.6%)
Sexual abuse	12,173 (17.4%)	60 (22.9%)	4,085 (7.2%)	44 (17.1%)
ACE group				
0	26,772 (38.2%)	67 (25.6%)	22,217 (38.9%)	78 (30.4%)
1–2	24,689 (35.2%)	88 (33.6%)	21,842 (38.3%)	66 (25.7%)
3+	18,681 (26.6%)	107 (40.8%)	13,013 (22.8%)	113 (44.0%)
Depression				
No	53,184 (75.8%)	144 (55.0%)	49,074 (86.0%)	154 (59.9%)
Yes	16,958 (24.2%)	118 (45.0%)	7,998 (14.0%)	103 (40.1%)

ACE, adverse childhood experiences.

### Effect measure modification among adults assigned female at birth

Table 3 shows the effect measure modification of ACE on depression by transgender identity among AFAB participants. Increasing ACE were associated with increasing prevalence of depression in both cisgender and transgender participants. Among cisgender participants, those with 1–2 and 3+ ACE had 1.84 times [PRR: 1.84 (95% CI 1.69–2.00)] and 3.14 times [PRR: 3.14 (95% CI 2.90–3.41)] the rate of depression compared to those with zero ACE, respectively. Among transgender participants, those with 1–2 and 3+ ACE had 3.23 times [PRR: 3.23 (95% CI 1.53–6.83)] and 4.65 times [4.65 (95% CI 2.06–10.47)] the rate of depression compared to those with zero ACE, respectively. Among AFAB participants with 0, 1–2, and 3+ ACE, the prevalence rate of depression for transgender individuals was 1.23 (PRR: 1.23 [95% CI 0.62–2.47), 2.17 [PRR: 2.17 (95% CI 1.62–2.91)], and 1.82 [PRR: 1.82 (95% CI 1.19–2.81)] times that of their cisgender counterparts.

**Table 3:** Modification of the association between adverse childhood experiences and depression by transgender identity among participants of the Behavioral Risk Factor Surveillance System 2019 and 2020 surveys who were assigned female at birth.

	PRRs (95% CI)			PRR (95% CI) for within strata of transgender identity	
	0 ACE	1–2 ACE	3+ ACE	1–2 vs. 0 ACE	3+ vs. 0 ACE
Cisgender	1 (Reference)	1.84 (1.69–2.00)	3.14 (2.90–3.41)	1.84 (1.69–2.00)	3.14 (2.90–3.41)
Transgender	1.23 (0.62–2.47)	3.98 (2.95–5.38)	5.73 (3.70–8.88)	3.23 (1.53–6.83)	4.65 (2.06–10.47)
PRR (95% CI) for within strata of ACE	1.23 (0.62–2.47)	2.17 (1.62–2.91)	1.82 (1.19–2.81)		

PRR, prevalence rate ratio; ACE, adverse childhood experiences; CI, confidence interval.

PRRs are adjusted for age, race, education, marital status, income, and employment.

Measure of effect measure modification on the multiplicative scale: ratio of PRRs (95% CI). 1–2 vs. 0 ACE: 1.76 (0.83–3.74); *p* = 0.141. 3 + vs. 0 ACE: 1.48 (0.65–3.34); *p* = 0.347.

Measure of effect measure modification on the additive scale: relative excess risk due to interaction (95% CI). 1–2 vs. 0 ACE: 1.91 (0.47–3.36); *p* = 0.009. 3+ vs. 0 ACE: 2.36 (−0.26–4.97); *p* = 0.077.

There was evidence of positive EMM by transgender identity on the additive scale in AFAB participants for 1–2 ACE vs. zero ACE [RERI: 1.91 (95% CI 0.47–3.36)]. This estimate indicates that AFAB participants with 1–2 ACE who identify as transgender may have higher combined risk of depression than the sum of the independent risks from having 1–2 ACE and transgender identity. This finding is reflected in the AFAB graph in Supplementary Figure S2. The slopes of the lines connecting predicted probabilities of depression for zero ACE and 1–2 ACE differ between cisgender and transgender groups. Transgender AFAB individuals had a steeper trend in predicted probabilities, i.e., a greater increase in predicted probabilities, than cisgender AFAB individuals.

### Effect measure modification among adults assigned male at birth

Table 4 shows the effect measure modification of ACE on depression by transgender identity among AMAB participants. Cisgender AMAB participants with 1–2 and 3+ ACE had 1.80 [PRR: 1.80 (95% CI 1.59–2.05)] and 3.34 [PRR: 3.34 (95% CI 2.95–3.78)] times the prevalence rate of depression compared to cisgender AMAB participants with zero ACE. Transgender AMAB participants with 1–2 and 3+ ACE had 0.87 [PRR: 0.87 (95% CI 0.30–2.51)] and 1.96 [PRR: 1.96 (95% CI 1.00–3.87)] times the prevalence rate of depression compared to transgender AMAB participants with zero ACE. Among AMAB participants with 0, 1–2, and 3+ ACE, the prevalence rate of depression for transgender individuals was 3.51 [PRR: 3.51 (95% CI 1.91–6.43)], 1.70 [PRR: 1.70 (95% CI 0.70–4.08)], and 2.06 [PRR: 2.06 (95% CI 1.49–2.85)] times that of their cisgender counterparts. There was no evidence of significant EMM on the multiplicative or additive scales among AMAB participants.

**Table 4:** Modification of the association between adverse childhood experiences and depression by transgender identity among participants of the Behavioral Risk Factor Surveillance System 2019 and 2020 surveys who were assigned male at birth.

	PRRs (95% CI)			PRR (95% CI) for within strata of transgender identity	
	0 ACE	1–2 ACE	3+ ACE	1–2 vs. 0 ACE	3+ vs. 0 ACE
Cisgender	1 (Reference)	1.80 (1.59–2.05)	3.34 (2.95–3.78)	1.80 (1.59–2.05)	3.34 (2.95–3.78)
Transgender	3.51 (1.91–6.43)	3.06 (1.27–7.38)	6.88 (4.90–9.68)	0.87 (0.30–2.51)	1.96 (1.00–3.87)
PRR (95% CI) for within strata of ACE	3.51 (1.91–6.43)	1.70 (0.70–4.08)	2.06 (1.49–2.85)		

PRR, prevalence rate ratio; ACE, adverse childhood experiences; CI, confidence interval.

PRRs are adjusted for age, race, education, marital status, income, and employment.

Measure of effect measure modification in the multiplicative scale: ratio of PRRs (95% CI). 1–2 vs. 0 ACE: 0.48 (0.17–1.40); *p* = 0.181. 3+ vs. 0 ACE: 0.59 (0.30–1.17); *p* = 0.129.

Measure of effect measure modification in the additive scale: Relative excess risk due to interaction (95% CI). 1–2 vs. 0 ACE: −1.25 (−4.67 to 2.15); *p* = 0.471. 3+ vs. 0 ACE: 1.04 (−2.01 to 4.09); *p* = 0.505.

## Discussion

We aimed to estimate whether the effect of ACE on depression was modified by transgender identity and whether this effect modification held regardless of adults’ experiences in childhood related to their assigned sex at birth (e.g., hormonal influences and sexism). We found a graded relationship between ACE and depression in both transgender and cisgender participants, which is consistent with prior literature (6, 8). However, transgender AFAB individuals had stronger associations between ACE and depression than their cisgender counterparts. Specifically, the results indicated a significant effect modification by transgender identity on the association between 1–2 ACE and depression among AFAB participants. Specifically, transgender AFAB adults with 1–2 ACE had a combined risk of depression that was higher than the sum of the two risks alone, i.e., identifying as transgender only or having 1–2 ACE only. The prevalence of depression among AFAB transgender adults was more than double that of AFAB cisgender adults. Efforts to reduce this disparity may benefit from targeting ACE. Additionally, AFAB transgender adults may have a greater need for childhood trauma-informed programs to address their depression than their cisgender female counterparts (29).

We hypothesize the mechanism behind these results involves the intersectionality of sexism, transphobia, and childhood adversities. Transgender AFAB adults could have experienced household abuse motivated by sexism and transphobia. Additionally, transgender AFAB adults could have experienced sexism and transphobia in their communities and received little to no support at home due to household dysfunction. These experiences could potentially increase emotional dysregulation and lead to poor mental health (30).

Transgender identity was strongly associated with depression in AMAB participants with zero ACE. This strong association may have led to the findings of non-significant effect modification of the ACE–depression association among AMAB participants. Patriarchal beliefs and toxic masculinity strongly stigmatize individuals assigned male at birth who display any feminine characteristics (31). As such, transgender AMAB individuals face high rates of victimization, hate crimes, and sexual abuse (32). These injustices may have such a strong effect on depression that the additional effect of experiencing household dysfunction and abuses may not be detectable with our data.

### Strengths

This was the largest study of the intersectional effect of ACE, transgender identity, and assigned sex on adult depression in the USA. To our knowledge, this was the first study of the differing effects of ACE on depression by transgender identity and sex assigned at birth. In addition to including transgender women and men, we included non-binary individuals, a population often left out of transgender research. We used data from the BRFSS, a well-established survey with defined procedures for data collection. Our survey-weighted analysis allowed our results to be generalizable to adults residing in the 16 US states that used the ACE and SOGI modules.

### Limitations

Our study had numerous limitations. The BRFSS 2019 and 2020 datasets only contained transgender identity and ACE information from 16 states, limiting our sample size and the generalizability of our results to other US states. Transgender identity could be underestimated due to hesitancy to disclose gender identity over the phone. As this study used ACE questions available in the BRFSS 2019 and 2020 questionnaires, we did not have information on specific adversities faced by transgender children that are not captured by the module. Transgender children are at higher risk of being kicked out of their homes, being rejected by their parents and peers, and experiencing hate crimes/harassment (33). We were unable to assess the effect of these ACE on depression in transgender adults.

Depression was reported as whether participants were ever told they had depression. It is possible depression was diagnosed in childhood, prior to ACE. Diagnosis of depression depends on both access to healthcare and willingness to seek care. The participants who reported they did not have depression can be categorized into three types: (1) participants truly without depression, (2) participants with depression who did not seek diagnosis, and (3) participants with depression who wanted to seek diagnosis, but had no healthcare access. Adults with higher ACE have lower rates of healthcare utilization and access (34). Thus, in our sample, adults with ACE are less likely to have healthcare access and seek a depression diagnosis. This misclassification of depression, which differs between participants with and without ACE, would reduce the estimated association between ACE and depression. Removing this misclassification would produce larger prevalence ratios than observed in this study.

### Policy implications

Our study highlights the need for interventions and policies targeted at improving the mental health of transgender adults. Examples of such policy include improvements in healthcare access through expansion of Medicare or Medicaid (35), decriminalization of drug use (36), increase in state funding to public benefit programs (37), and promotion of LGBTQ+ safety in schools (38). These policies range from targeting ACE in childhood for primary prevention to screening, diagnosing, and treating adulthood depression with secondary and tertiary prevention measures.

Primary prevention focuses on reducing ACE and creating supportive environments at home and school. Giving parents access to free, unconditional health insurance and reducing drug-related stigma and incarceration may help them seek care for mental illness and substance dependency. This could potentially lead to a reduction in household dysfunction. Additionally, state-funded public benefit programs (e.g., housing support and child-care support) significantly reduce child maltreatment (37). A systematic review showed school safety as a protective factor against self-harm in transgender students (39). Promoting LGBTQ+ safety at school includes establishing inclusive policies and protections against LGBTQ+ targeted bullying, school staff training on LGBTQ+ identities, LGBTQ+-led student clubs, and LGBTQ+-inclusive school curricula and textbooks (38).

Secondary and tertiary prevention focuses on screening, diagnosing, and alleviating depression to prevent further harm. Transgender adults having access to health insurance improves their ability to seek out trauma-informed mental health services and gender-affirming care (e.g., hormone replacement therapy and gender confirmation surgery). A systematic review showed hormone replacement therapy significantly reduced depression and other mental illnesses in transgender adults (40).

### Research implications

Further research should examine whether transgender identity modifies the association of ACE with other mental illnesses of importance to the transgender community, such as anxiety, PTSD, and suicidal ideation. More states should include the ACE and SOGI modules in their annual BRFSS to enumerate ACE in their transgender population. The ACE module should be expanded to include ACE prevalent in the transgender population, e.g., homelessness, gender-based harassment, and parental/peer rejection. Our study showed heterogeneity within the transgender population by assigned sex at birth. Researchers should collect and analyze data on ACE disaggregated by assigned sex and gender identity to account for the intersectionality between sexism, transphobia, and ACE and their combined impact on transgender health.

Definitions of transgender identity and gender often vary in the literature (41). Having clear, universal definitions actively used in research would increase discoveries of significant sub-population heterogeneity. Additionally, gender is a spectrum, and transgender identity includes a range of experiences from transgender men, women, non-binary, and genderqueer individuals (42). Defining gender identity through questions about gender expression, self-identity, and norms instead of simply categorizing participants into three or more gender labels can provide more information about their experiences in society, and by extension their health.

## Conclusion

Transgender adults who faced childhood adversities may benefit more from childhood trauma-informed interventions to treat their depression than their cisgender counterparts. This may be particularly applicable to transgender adults who were assigned female at birth. There is a need to expand data collection of ACE, transgender identity, and experiences (both biological and social) associated with sex assigned at birth. These data, along with improved survey questions on gender identity and assigned sex, will improve our understanding of the intersecting influences of sexism, transphobia, and ACE on adult mental health. Additionally, these data will allow for the identification of particularly vulnerable subpopulations in need of greater support. Future studies should consider if transgender identity modifies the associations between ACE and other mental health conditions prevalent in the transgender community (e.g., anxiety disorder, PTSD, suicidal ideation).

## Data Availability

The original contributions presented in the study are included in the article/Supplementary Materials, further inquiries can be directed to the corresponding author.
