# Supplementary figures and images for: The interaction of adverse childhood experiences, sex, and transgender identity as risk factors for depression: disparities in transgender adults
Source: Front Glob Womens Health. 2024 Dec 24;5:1306065. doi: 10.3389/fgwh.2024.1306065 (PMC11703960; doi:10.3389/fgwh.2024.1306065)

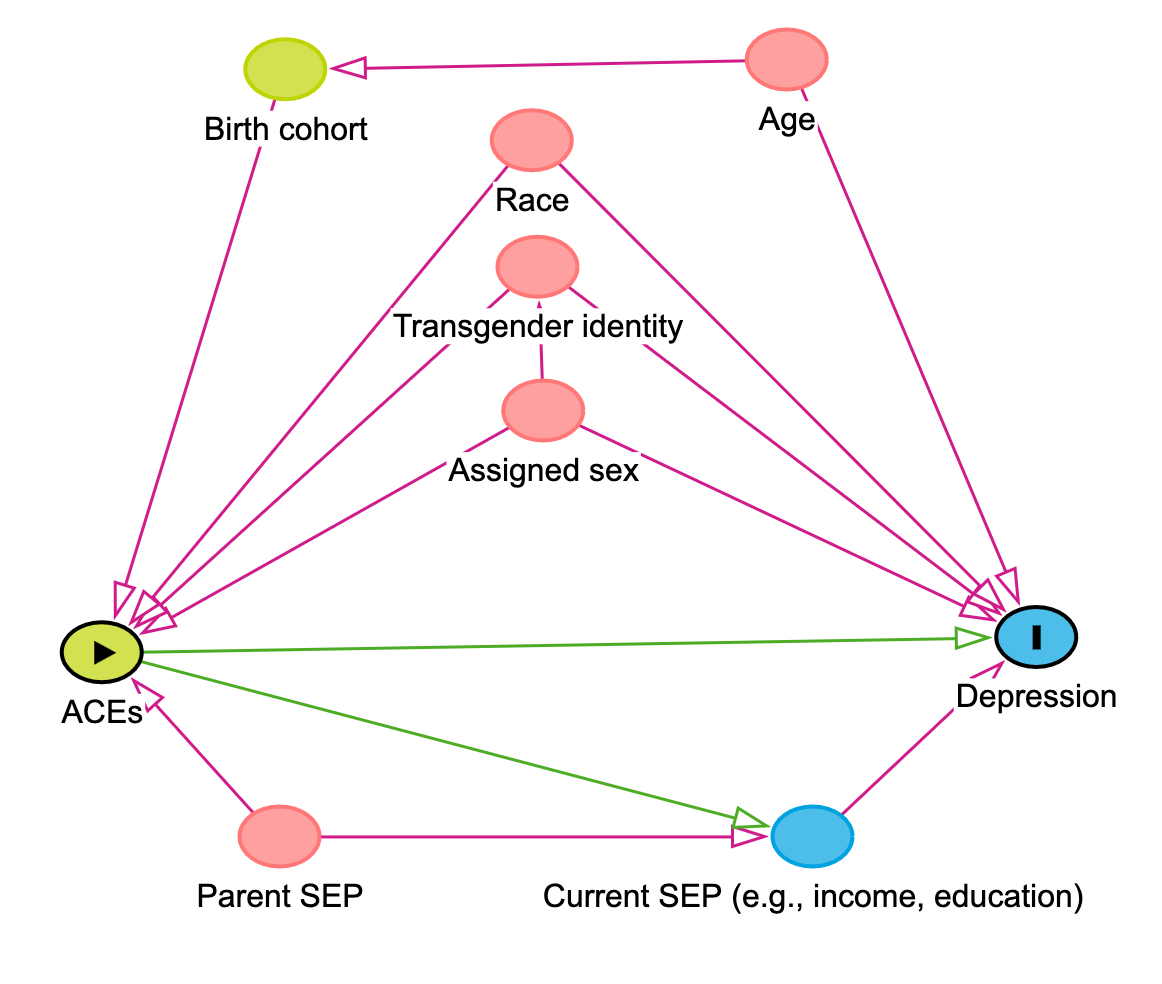

Supplement: Supplementary file 1 [file Image1.tiff]

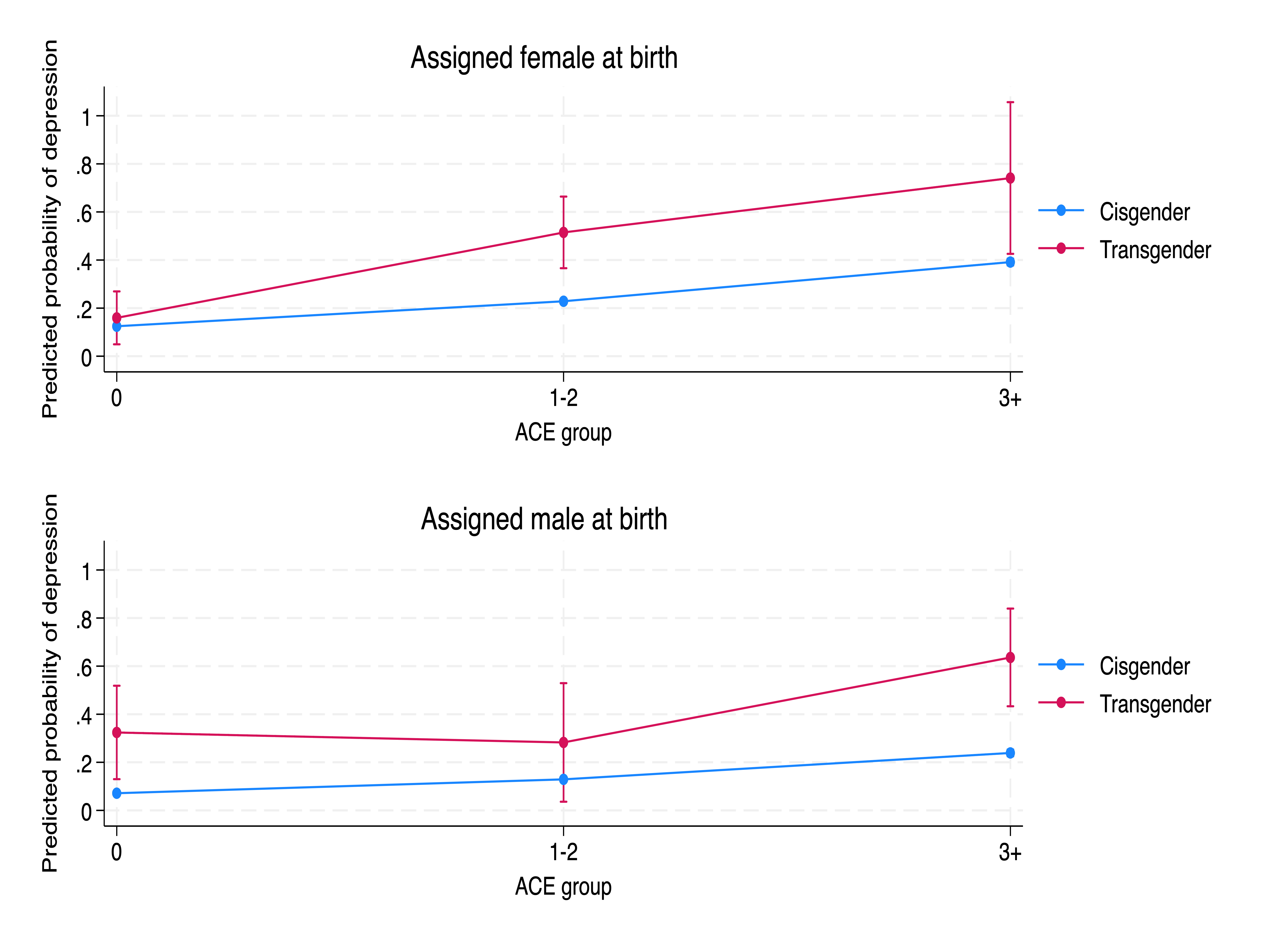

Supplement: Supplementary file 2 [file Image2.jpeg]
