# Supplementary material for: The interaction of adverse childhood experiences, sex, and transgender identity as risk factors for depression: disparities in transgender adults
Source: Front Glob Womens Health. 2024 Dec 24;5:1306065. doi: 10.3389/fgwh.2024.1306065 (PMC11703960; doi:10.3389/fgwh.2024.1306065)
Supplement: Supplementary file 3 [file Table1.docx]

**SUPPLEMENT**

**Figure S1**

Directed acyclic graph showing the presumed relationship between adverse childhood experiences, depression, transgender identity, and other demographic factors.

**Figure S2**

Predicted probabilities of depression with 95% confidence intervals by adverse childhood experiences, transgender identity, and assigned sex at birth. Models were adjusted for age, race, income, education, employment, and marital status. Differences in line slope between the transgender and cisgender groups indicate effect measure modification.

**Table S1**

Definitions and coding of adverse childhood experiences retrieved from the Behavioral Risk Factor Surveillance System 2019 and 2020 surveys.

| ACE | Question(s) | Response options | Coding in study |
| --- | --- | --- | --- |
| Mental illness in household | Did you live with anyone who was depressed, mentally ill, or suicidal? | Yes, No, Unsure, Refused, or Missing | 1 = Yes    0 = No, Unsure, Refused, or Missing |
| Alcoholic in household | Did you live with anyone who was a problem drinker or alcoholic? |  |  |
| Substance abuser in household | Did you live with anyone who used illegal street drugs or who abused prescription medications? |  |  |
| Family member incarcerated | Did you live with anyone who served time or was sentenced to serve time in a prison, jail, or other correctional facility? |  |  |
| Parental divorce or separation | Were your parents separated or divorced? | Yes, No, Not Married, Unsure, Refused, or Missing | 1 = Yes  0 = No, not married, Unsure, Refused, or Missing |
| Domestic violence in household | How often did your parents or adults in your home ever slap, hit, kick, punch or beat each other up? | Never, Once, More than once, Unsure, Refused, or Missing | 1 = Once or More than once  0 = Never, Unsure, Refused, or Missing |
| Physical abuse | Before age 18, how often did a parent or adult in your home ever hit, beat, kick, or physically hurt you in any way? Do not include spanking. Would you say— |  |  |
| Verbal abuse | How often did a parent or adult in your home ever swear at you, insult you, or put you down? |  |  |
| Sexual abuse | How often did anyone at least 5 years older than you or an adult, ever touch you sexually? | Never, Once, More than once, Unsure, Refused, or Missing | 1 = Once or More than once in at least one question  0 = Never, Unsure, Refused, or missing in all questions |
|  | How often did anyone at least 5 years older than you or an adult, try to make you touch sexually? |  |  |
|  | How often did anyone at least 5 years older than you or an adult, force you to have sex? |  |  |

^a^ACE = adverse childhood experiences.

^b^Information was retrieved from the 2019 and 2020 Behavioral Risk Factor Surveillance System adverse childhood experiences modules.

**Table S2**

Modification of the association between adverse childhood experiences and depression by transgender identity among participants of the Behavioral Risk Factor Surveillance System 2019 and 2020 surveys who were assigned female at birth.

|  | PRRs (95% CI) | | |  | PRR (95% CI) for within strata of transgender identity | |
| --- | --- | --- | --- | --- | --- | --- |
|  | 0 ACE | 1-2 ACE | 3+ ACE |  | 1-2 vs. 0 ACE | 3+ vs. 0 ACE |
| Cisgender | 1 (Reference) | 1.84 (1.69-2.01) | 3.35 (3.09-3.63) |  | 1.84 (1.69-2.01) | 3.35 (3.09-3.63) |
| Transgender | 1.42 (0.74-2.75) | 4.15 (3.09-5.56) | 5.88 (4.27-8.11) |  | 2.91 (1.43-5.93) | 4.13 (2.00-8.52) |
| PRR (95% CI) for within strata of ACE: transgender vs. cisgender | 1.42 (0.74-2.75) | 2.25 (1.69-3.00) | 1.76 (1.28-2.41) |  |  |  |

^a^PRR = prevalence rate ratio. N = number. ACE = adverse childhood experiences. CI = confidence interval.

^b^PRRs are adjusted for age and race.

^c^Measure of effect measure modification on the multiplicative scale: ratio of PRRs (95% CI).

1-2 vs 0 ACE: 1.58 (0.77-3.23); p=0.212

3+ vs 0 ACE: 1.23 (0.60-2.55); p=0.572

^d^Measure of effect measure modification on the additive scale: Relative excess risk due to interaction (95% CI).

1-2 vs 0 ACE: 1.88 (0.37-3.38); p=0.014

3+ vs 0 ACE: 2.11 (0.04-4.18); p=0.045

**Table S3**

Modification of the association between adverse childhood experiences and depression by transgender identity among participants of the Behavioral Risk Factor Surveillance System 2019 and 2020 surveys who were assigned male at birth.

|  | PRRs (95% CI) | | |  | PRR (95% CI) for within strata of transgender identity | |
| --- | --- | --- | --- | --- | --- | --- |
|  | 0 ACE | 1-2 ACE | 3+ ACE |  | 1-2 vs. 0 ACE | 3+ vs. 0 ACE |
| Cisgender | 1 (Reference) | 1.87 (1.65-2.12) | 3.77 (3.34-4.26) |  | 1.87 (1.65-2.12) | 3.77 (3.34-4.26) |
| Transgender | 3.98 (2.11-7.49) | 5.03 (2.47-10.24) | 9.41 (7.14-12.39) |  | 1.26 (0.49-3.24) | 2.36 (1.20-4.64) |
| PRR (95% CI) for within strata of ACE: transgender vs. cisgender | 3.98 (2.11-7.49) | 2.69 (1.32-5.45) | 2.49 (1.93-3.23) |  |  |  |

^a^PRR = prevalence rate ratio. ACE = adverse childhood experiences. CI = confidence interval.

^b^PRRs are adjusted for age and race.

^c^Measure of effect measure modification on the multiplicative scale: ratio of PRRs (95% CI).

1-2 vs 0 ACE: 0.68 (0.26-1.74); p=0.417

3+ vs 0 ACE: 0.63 (0.32-1.24); p=0.180

^d^Measure of effect measure modification on the additive scale: Relative excess risk due to interaction (95% CI).

1-2 vs 0 ACE: 0.18 (-4.15-4.50); p=0.936

3+ vs 0 ACE: 2.66 (-0.79-6.11); p=0.131
