# Supplementary material for: The interaction of adverse childhood experiences, sex, and transgender identity as risk factors for depression: disparities in transgender adults
Source: Front Glob Womens Health. 2024 Dec 24;5:1306065. doi: 10.3389/fgwh.2024.1306065 (PMC11703960; doi:10.3389/fgwh.2024.1306065)
Supplement: Supplementary file 4 [file Table2.docx]

********************************************************************************

********************************************************************************

* Importing and merging datasets

* 2019

import sasxport5 "PATH/BRFSS2019", replace

keep acedeprs acedivrc acedrink acedrugs acehurt1 acehvsex aceprisn acepunch aceswear acetouch acetthem addepev3 trnsgndr _sex _age_g _imprace _ststr _psu _llcpwt _incomg _educag employ1 marital _state idate _urbstat

keep if inlist(_state, 10, 12, 19, 28, 44, 45, 47, 51, 54, 55) //10 - 342,781

save "PATH/data19", replace

* 2020

import sasxport5 "PATH/BRFSS2020", replace

keep acedeprs acedivrc acedrink acedrugs acehurt1 acehvsex aceprisn acepunch aceswear acetouch acetthem addepev3 trnsgndr _sex _age_g _imprace _ststr _psu _llcpwt _incomg _educag employ1 marital _state idate _urbstat

keep if inlist(_state, 12, 13, 15, 16, 19, 30, 44, 45, 48, 49, 51, 55) //12 - 305,345

save "PATH/data20", replace

append using "PATH/data19"

save "PATH/data1920", replace

********************************************************************************

********************************************************************************

* MAIN ANALYSIS

********************************************************************************

********************************************************************************

* Start data editing

use "PATH/data1920", replace

* Drop missing trans, depression, and all aces

drop if acedeprs>3 & acedivrc>3 & acedrink>3 & acedrugs>3 & acehurt1>3 & acehvsex>3 & aceprisn>3 & acepunch>3 & aceswear>3 & acetouch>3 & acetthem>3

drop if addepev3>2

drop if trnsgndr>4

drop if _educag==9

drop if marital==9 | marital==.

drop if employ1==9 | employ1==.

count // 127,733

* Creating new variables

** New weight = half of old weight

gen _llcpwt2 = _llcpwt/2

** Trans binary variable

gen transbin = .

replace transbin=1 if trnsgndr==1 | trnsgndr==2 | trnsgndr==3

replace transbin=0 if trnsgndr==4

** Recode depression to (1/0)

recode addepev3 2=0

** Sex assigned at birth (Male=1, Female=0)

recode _sex 2=0

gen birthsex=.

replace birthsex = _sex if trnsgndr==3 | trnsgndr==4

replace birthsex = 1 if trnsgndr==1

replace birthsex = 0 if trnsgndr==2

** ACE -> ACE count -> ACE three groups (0, 1-2, 3+)

recode acedeprs acedivrc acedrink acedrugs aceprisn (2 7 8 9 . = 0)

recode acehurt1 acehvsex acepunch aceswear acetouch acetthem (1 7 9 . = 0) (2 3 = 1)

gen acesexany = 0

replace acesexany=1 if acetouch==1 | acehvsex==1 | acetthem==1

gen acecount = acedeprs + acedivrc + acedrink + acedrugs + aceprisn + acehurt1 +acepunch + aceswear + acesexany

gen acegrp=.

replace acegrp=0 if acecount==0

replace acegrp=1 if acecount==1 | acecount==2

replace acegrp=2 if acecount>2

**Income groups

tab _incomg, m

**Education

tab _educag, m

**Marital

tab marital, m

recode marital (2 3 4 5 6 = 0)

**Employment

tab employ1, m

recode employ1 (1 2 = 1) (3 4 = 2) (5 6 7 8 = 3)

** Labels

label var transbin "Gender identity"

label var addepev3 "Depression"

label var acedeprs "Mental illness in household"

label var acedrink "Alcoholic in household"

label var acedrugs "Substance abuser in household"

label var acedivrc "Parental divorce or seperation"

label var aceprisn "Family member incarcerated"

label var acepunch "Domestic violence in household"

label var acehurt1 "Physical abuse"

label var aceswear "Verbal abuse"

label var acesexany "Sexual abuse"

label var acegrp "ACE group"

label var birthsex "Assigned sex"

label var _age_g "Age group"

label var _imprace "Race"

label var _incomg "Income group"

label var _educag "Education"

label var marital "Married"

label var employ1 "Employment"

**Label values

label define yesno 1 "Yes" 0 "No"

label values addepev3 acedeprs acedrink acedrugs acedivrc aceprisn acepunch acehurt1 aceswear acesexany marital yesno

*Trans

label define trans 1 "Transgender" 0 "Cisgender"

label values transbin trans

*Ace group

label define acelab 0 "0" 1 "1-2" 2 "3+"

label values acegrp acelab

*Birthsex

label define sex 1 "Assigned male at birth" 0 "Assigned female at birth"

label values birthsex sex

*Age

label define age 1 "18-24" 2 "25-34" 3 "35-44" 4 "45-54" 5 "55-64" 6 "65+"

label values _age_g age

*Race

label define race 1 "Non-Hispanic White" 2 "Non-Hispanic Black" 3 "Non-Hispanic Asian" 4 "Non-Hispanic American Indian/Alaskan Native" 5 "Hispanic" 6 "Non-Hispanic Other"

label values _imprace race

*Income group

label define income 1 "<$15,000" 2 "$15,000 to $24,999" 3 "$25,000 to $34,999" 4 "$35,000 to $49,999" 5 ">$50,000" 9 "Missing"

label values _incomg income

*Education

label define edu 1 "Less than high school graduate" 2 "High school graduate" 3 "Some college" 4 "College graduate"

label values _educag edu

*Employment

label define emp 1 "Employed" 2 "Unemployed" 3 "Other"

label values employ1 emp

save "PATH/data_final1", replace

********************************************************************************

********************************************************************************

* Table 2

collect clear

collect style header acedeprs acedrink acedrugs acedivrc aceprisn acepunch acehurt1 aceswear acesexany, level(hide)

collect style save hide-levels, replace

dtable i.birthsex i._age_g i._imprace i._educag i.marital i._incomg i.employ1 1.acedeprs 1.acedrink 1.acedrugs 1.acedivrc 1.aceprisn 1.acepunch 1.acehurt1 1.aceswear 1.acesexany i.acegrp i.addepev3, by(transbin)

collect style use hide-levels, override

collect preview

collect export "table2.docx", replace

* Table 3

gen group=.

replace group=1 if birthsex==0 & transbin==0

replace group=2 if birthsex==0 & transbin==1

replace group=3 if birthsex==1 & transbin==0

replace group=4 if birthsex==1 & transbin==1

collect clear

collect style header acedeprs acedrink acedrugs acedivrc aceprisn acepunch acehurt1 aceswear acesexany, level(hide)

collect style save hide-levels, replace

dtable i._age_g i._imprace i._educag i.marital i._incomg i.employ1 1.acedeprs 1.acedrink 1.acedrugs 1.acedivrc 1.aceprisn 1.acepunch 1.acehurt1 1.aceswear 1.acesexany i.acegrp i.addepev3, by(group)

collect style use hide-levels, override

collect preview

collect export "table3.docx", replace

********************************************************************************

*******************************************************************************

use "PATH/data_final1", replace

* Survey weights, etc.

svyset, clear

svyset _psu [weight=_llcpwt2], strata(_ststr) singleunit(centered)

*format date

gen date2 = date(idate, "MDY")

*Recode income group

recode _incomg (9=.)

* Model

mi set mlong

mi register imputed _incomg

set more off

mi impute chained (mlogit) _incomg = i._age_g i._imprace i._educag i.marital i.employ1 i._state i._urbstat date2 [pweight=_llcpwt2], add(20) rseed (54321)

tab _mi_m

* # includes only the interaction terms

set more off

mi estimate, post: svy: glm addepev3 i.acegrp#i.transbin i._age_g i._imprace i._educag i.marital i._incomg i.employ1 if birthsex==0, family(poisson) link(log)

***********************

*TABLE ANALYSIS STARTS*

***********************

* Ref=Cis w/ zero ACE (00) (5)

* Cis

lincom 0.transbin#1.acegrp - 0.transbin#0.acegrp, or /*01 vs 00*/

lincom 0.transbin#2.acegrp - 0.transbin#0.acegrp, or /*02 vs 00*/

* Trans

lincom 1.transbin#0.acegrp - 0.transbin#0.acegrp, or /*10 vs 00*/

lincom 1.transbin#1.acegrp - 0.transbin#0.acegrp, or /*11 vs 00*/

lincom 1.transbin#2.acegrp - 0.transbin#0.acegrp, or /*12 vs 00*/

* Within strata of ACE

lincom 1.transbin#0.acegrp - 0.transbin#0.acegrp, or /*10 vs 00*/

lincom 1.transbin#1.acegrp - 0.transbin#1.acegrp, or /*11 vs 01*/

lincom 1.transbin#2.acegrp - 0.transbin#2.acegrp, or /*12 vs 02*/

* Within strata of Cis and Trans (6)

* Cis

lincom 0.transbin#1.acegrp - 0.transbin#0.acegrp, or /*01 vs 00*/

lincom 0.transbin#2.acegrp - 0.transbin#0.acegrp, or /*02 vs 00*/

* Trans

lincom 1.transbin#1.acegrp - 1.transbin#0.acegrp, or /*11 vs 10*/

lincom 1.transbin#2.acegrp - 1.transbin#0.acegrp, or /*12 vs 10*/

* Multipliplicative interaction

* 1-2 vs 0 ACE

lincom 1.transbin#1.acegrp - 1.transbin#0.acegrp - 0.transbin#1.acegrp + 0.transbin#0.acegrp, or

* 3+ vs 0 ACE

lincom 1.transbin#2.acegrp - 1.transbin#0.acegrp - 0.transbin#2.acegrp + 0.transbin#0.acegrp, or

* Additive interaction

set more off

mi estimate, post: svy: glm addepev3 i.acegrp##i.transbin i._age_g i._imprace i._educag i.marital i._incomg i.employ1 if birthsex==0, family(poisson) link(log)

* 1-2 vs 0 ACE

nlcom exp(_b[1.transbin] + _b[1.acegrp] + _b[1.transbin#1.acegrp]) - exp(_b[1.acegrp]) - exp(_b[1.transbin]) + 1

* 3+ vs 0 ACE

nlcom exp(_b[1.transbin] + _b[2.acegrp] + _b[1.transbin#2.acegrp]) - exp(_b[2.acegrp]) - exp(_b[1.transbin]) + 1

* predicted probabilities for the different groups

mimrgns, by(transbin) at(acegrp=(0 1 2)) cmdmargins expression(exp(predict(xb)))

marginsplot //Save as "PATH/FigureS2 AFAB.gph"

********************************************************************************

********************************************************************************

* Table 5: AMAB

* Model

set more off

mi estimate, post: svy: glm addepev3 i.acegrp#i.transbin i._age_g i._imprace i._educag i.marital i._incomg i.employ1 if birthsex==1, family(poisson) link(log)

***********************

*TABLE ANALYSIS STARTS*

***********************

* Ref=Cis w/ zero ACE (0,0) (5)

* Cis

lincom 0.transbin#1.acegrp - 0.transbin#0.acegrp, or /*01 vs 00*/

lincom 0.transbin#2.acegrp - 0.transbin#0.acegrp, or /*02 vs 00*/

* Trans

lincom 1.transbin#0.acegrp - 0.transbin#0.acegrp, or /*10 vs 00*/

lincom 1.transbin#1.acegrp - 0.transbin#0.acegrp, or /*11 vs 00*/

lincom 1.transbin#2.acegrp - 0.transbin#0.acegrp, or /*12 vs 00*/

* Within strata of ACE

lincom 1.transbin#0.acegrp - 0.transbin#0.acegrp, or /*10 vs 00*/

lincom 1.transbin#1.acegrp - 0.transbin#1.acegrp, or /*11 vs 01*/

lincom 1.transbin#2.acegrp - 0.transbin#2.acegrp, or /*12 vs 02*/

* Within Strata of Cis and Trans (6)

* Cis

lincom 0.transbin#1.acegrp - 0.transbin#0.acegrp, or /*01 vs 00*/

lincom 0.transbin#2.acegrp - 0.transbin#0.acegrp, or /*02 vs 00*/

* Trans

lincom 1.transbin#1.acegrp - 1.transbin#0.acegrp, or /*11 vs 10*/

lincom 1.transbin#2.acegrp - 1.transbin#0.acegrp, or /*12 vs 10*/

* Multipliplicative interaction

* 1-2 vs 0 ACE

lincom 1.transbin#1.acegrp - 1.transbin#0.acegrp - 0.transbin#1.acegrp + 0.transbin#0.acegrp, or

* 3+ vs 0 ACE

lincom 1.transbin#2.acegrp - 1.transbin#0.acegrp - 0.transbin#2.acegrp + 0.transbin#0.acegrp, or

* Additive interaction

* # includes only the interaction terms

set more off

mi estimate, post: svy: glm addepev3 i.acegrp##i.transbin i._age_g i._imprace i._educag i.marital i._incomg i.employ1 if birthsex==1, family(poisson) link(log)

* 1-2 vs 0 ACE

nlcom exp(_b[1.transbin] + _b[1.acegrp] + _b[1.transbin#1.acegrp]) - exp(_b[1.acegrp]) - exp(_b[1.transbin]) + 1

* 3+ vs 0 ACE

nlcom exp(_b[1.transbin] + _b[2.acegrp] + _b[1.transbin#2.acegrp]) - exp(_b[2.acegrp]) - exp(_b[1.transbin]) + 1

* predicted probabilities for the different groups

mimrgns, by(transbin) at(acegrp=(0 1 2)) cmdmargins expression(exp(predict(xb)))

marginsplot //Save as "PATH/FigureS2 AMAB.gph"

********************************************************************************

********************************************************************************

* FIGURE S2

graph combine "PATH/FigureS2 AFAB.gph" "PATH/FigureS2 AMAB.gph", xcommon ycommon rows(2)

********************************************************************************

********************************************************************************

* SUPPLEMENTAL ANALYSIS

********************************************************************************

********************************************************************************

* Start data editing

use "PATH/data1920", replace

* Drop missing trans, depression, and all aces

drop if trnsgndr>4

drop if addepev3>2

drop if acedeprs>3 & acedivrc>3 & acedrink>3 & acedrugs>3 & acehurt1>3 & acehvsex>3 & aceprisn>3 & acepunch>3 & aceswear>3 & acetouch>3 & acetthem>3

count // 129,446

* Creating new variables

** New weight = half of old weight

gen _llcpwt2 = _llcpwt/2

** Trans binary variable

gen transbin = .

replace transbin=1 if trnsgndr==1 | trnsgndr==2 | trnsgndr==3

replace transbin=0 if trnsgndr==4

** Recode depression to (1/0)

recode addepev3 2=0

** Sex assigned at birth (Male=1, Female=0)

recode _sex 2=0

gen birthsex=.

replace birthsex = _sex if trnsgndr==3 | trnsgndr==4

replace birthsex = 1 if trnsgndr==1

replace birthsex = 0 if trnsgndr==2

** ACE -> ACE count -> ACE three groups (0, 1-2, 3+)

recode acedeprs acedivrc acedrink acedrugs aceprisn (2 7 8 9 . = 0)

recode acehurt1 acehvsex acepunch aceswear acetouch acetthem (1 7 9 . = 0) (2 3 = 1)

gen acesexany = 0

replace acesexany=1 if acetouch==1 | acehvsex==1 | acetthem==1

gen acecount = acedeprs + acedivrc + acedrink + acedrugs + aceprisn + acehurt1 +acepunch + aceswear + acesexany

gen acegrp=.

replace acegrp=0 if acecount==0

replace acegrp=1 if acecount==1 | acecount==2

replace acegrp=2 if acecount>2

** Labels

label var transbin "Gender identity"

label var addepev3 "Depression (Yes/No)"

label var acedeprs "Mental illness in household"

label var acedrink "Alcoholic in household"

label var acedrugs "Substance abuser in household"

label var acedivrc "Parental divorce or seperation"

label var aceprisn "Family member incarcerated"

label var acepunch "Domestic violence in household"

label var acehurt1 "Physical abuse"

label var aceswear "Verbal abuse"

label var acesexany "Sexual abuse"

label var acegrp "ACE group"

label var birthsex "Sex assigned at birth"

label var _age_g "Age group"

label var _imprace "Race"

**Label values

label define yesno 1 "Yes" 0 "No"

label values addepev3 acedeprs acedrink acedrugs acedivrc aceprisn acepunch acehurt1 aceswear acesexany yesno

*Trans

label define trans 1 "Transgender" 0 "Cisgender"

label values transbin trans

*Ace group

label define acelab 0 "0" 1 "1-2" 2 "3+"

label values acegrp acelab

*Birthsex

label define sex 1 "Assigned male at birth" 0 "Assigned female at birth"

label values birthsex sex

*Age

label define age 1 "18-24" 2 "25-34" 3 "35-44" 4 "45-54" 5 "55-64" 6 "65+"

label values _age_g age

*Race

label define race 1 "Non-Hispanic White" 2 "Non-Hispanic Black" 3 "Non-Hispanic Asian" 4 "Non-Hispanic American Indian/Alaskan Native" 5 "Hispanic" 6 "Non-Hispanic Other"

label values _imprace race

save "PATH/data_final2", replace

********************************************************************************

********************************************************************************

* Table 4: AFAB

use "PATH/data_final2", replace

* Survey weights, etc.

svyset, clear

svyset _psu [weight=_llcpwt2], strata(_ststr) singleunit(centered)

* Model

svy: glm addepev3 i.acegrp#i.transbin i._age_g i._imprace if birthsex==0, family(poisson) link(log)

***********************

*TABLE ANALYSIS STARTS*

***********************

* Ref=Cis w/ zero ACE (00) (5)

* Cis

lincom 0.transbin#1.acegrp - 0.transbin#0.acegrp, or /*01 vs 00*/

lincom 0.transbin#2.acegrp - 0.transbin#0.acegrp, or /*02 vs 00*/

* Trans

lincom 1.transbin#0.acegrp - 0.transbin#0.acegrp, or /*10 vs 00*/

lincom 1.transbin#1.acegrp - 0.transbin#0.acegrp, or /*11 vs 00*/

lincom 1.transbin#2.acegrp - 0.transbin#0.acegrp, or /*12 vs 00*/

* Within strata of ACE

lincom 1.transbin#0.acegrp - 0.transbin#0.acegrp, or /*10 vs 00*/

lincom 1.transbin#1.acegrp - 0.transbin#1.acegrp, or /*11 vs 01*/

lincom 1.transbin#2.acegrp - 0.transbin#2.acegrp, or /*12 vs 02*/

* Within strata of Cis and Trans (6)

* Cis

lincom 0.transbin#1.acegrp - 0.transbin#0.acegrp, or /*01 vs 00*/

lincom 0.transbin#2.acegrp - 0.transbin#0.acegrp, or /*02 vs 00*/

* Trans

lincom 1.transbin#1.acegrp - 1.transbin#0.acegrp, or /*11 vs 10*/

lincom 1.transbin#2.acegrp - 1.transbin#0.acegrp, or /*12 vs 10*/

* Multipliplicative interaction

* 1-2 vs 0 ACE

lincom 1.transbin#1.acegrp - 1.transbin#0.acegrp - 0.transbin#1.acegrp + 0.transbin#0.acegrp, or

* 3+ vs 0 ACE

lincom 1.transbin#2.acegrp - 1.transbin#0.acegrp - 0.transbin#2.acegrp + 0.transbin#0.acegrp, or

* Additive interaction

svy: glm addepev3 i.acegrp##i.transbin i._age_g i._imprace if birthsex==0, family(poisson) link(log)

* 1-2 vs 0 ACE

nlcom exp(_b[1.transbin] + _b[1.acegrp] + _b[1.transbin#1.acegrp]) - exp(_b[1.acegrp]) - exp(_b[1.transbin]) + 1

* 3+ vs 0 ACE

nlcom exp(_b[1.transbin] + _b[2.acegrp] + _b[1.transbin#2.acegrp]) - exp(_b[2.acegrp]) - exp(_b[1.transbin]) + 1

********************************************************************************

********************************************************************************

* Table 5: AMAB

* Model

svy: glm addepev3 i.acegrp#i.transbin i._age_g i._imprace if birthsex==1, family(poisson) link(log)

***********************

*TABLE ANALYSIS STARTS*

***********************

* Ref=Cis w/ zero ACE (0,0) (5)

* Cis

lincom 0.transbin#1.acegrp - 0.transbin#0.acegrp, or /*01 vs 00*/

lincom 0.transbin#2.acegrp - 0.transbin#0.acegrp, or /*02 vs 00*/

* Trans

lincom 1.transbin#0.acegrp - 0.transbin#0.acegrp, or /*10 vs 00*/

lincom 1.transbin#1.acegrp - 0.transbin#0.acegrp, or /*11 vs 00*/

lincom 1.transbin#2.acegrp - 0.transbin#0.acegrp, or /*12 vs 00*/

* Within strata of ACE

lincom 1.transbin#0.acegrp - 0.transbin#0.acegrp, or /*10 vs 00*/

lincom 1.transbin#1.acegrp - 0.transbin#1.acegrp, or /*11 vs 01*/

lincom 1.transbin#2.acegrp - 0.transbin#2.acegrp, or /*12 vs 02*/

* Within Strata of Cis and Trans (6)

* Cis

lincom 0.transbin#1.acegrp - 0.transbin#0.acegrp, or /*01 vs 00*/

lincom 0.transbin#2.acegrp - 0.transbin#0.acegrp, or /*02 vs 00*/

* Trans

lincom 1.transbin#1.acegrp - 1.transbin#0.acegrp, or /*11 vs 10*/

lincom 1.transbin#2.acegrp - 1.transbin#0.acegrp, or /*12 vs 10*/

* Multipliplicative interaction

* 1-2 vs 0 ACE

lincom 1.transbin#1.acegrp - 1.transbin#0.acegrp - 0.transbin#1.acegrp + 0.transbin#0.acegrp, or

* 3+ vs 0 ACE

lincom 1.transbin#2.acegrp - 1.transbin#0.acegrp - 0.transbin#2.acegrp + 0.transbin#0.acegrp, or

* Additive interaction

svy: glm addepev3 i.acegrp##i.transbin i._age_g i._imprace if birthsex==1, family(poisson) link(log)

* 1-2 vs 0 ACE

nlcom exp(_b[1.transbin] + _b[1.acegrp] + _b[1.transbin#1.acegrp]) - exp(_b[1.acegrp]) - exp(_b[1.transbin]) + 1

* 3+ vs 0 ACE

nlcom exp(_b[1.transbin] + _b[2.acegrp] + _b[1.transbin#2.acegrp]) - exp(_b[2.acegrp]) - exp(_b[1.transbin]) + 1

********************************************************************************

********************************************************************************
